# Investigation of acute severe hepatitis in children: A review of liver transplant data, Canada, 2021–2022

**DOI:** 10.14745/ccdr.v49i06a01

**Published:** 2023-06-01

**Authors:** Vanessa Morton, Meghan Hamel, Vicky Ng, Susan Gilmour, Fernando Alvarez, Marina I Salvadori

**Affiliations:** 1 Centre for Food-borne, Environmental and Zoonotic Infectious Diseases, Public Health Agency of Canada, Guelph, ON; 2 Centre for Food-borne, Environmental and Zoonotic Infectious Diseases, Public Health Agency of Canada, Ottawa, ON; 3 SickKids Transplant Center, The Hospital for Sick Children, Toronto, ON; 4 Pediatrics, Stollery Children’s Hospital, Edmonton, AB; 5 Division of Paediatric Gastroenterology, Hospital Sainte-Justine, Montréal, QC; 6 Public Health Agency of Canada, Ottawa, ON; 7 Department of Pediatrics, McGill University, Montréal, QC

**Keywords:** hepatitis, paediatric, transplant, liver transplant, acute hepatitis, Canada

## Abstract

An increase in severe acute hepatitis of unknown etiology was first reported in the United Kingdom in April 2022. Following this report, the Public Health Agency of Canada connected with three paediatric liver transplant centres across Canada to determine if an increase in liver transplants was noted. Data demonstrated no observable increase in the number of transplants conducted in 2022. These data in conjunction with a federal, provincial, territorial investigation provided insight into the situation in Canada.

## Introduction

In April 2022, the World Health Organization was notified of 10 cases of severe acute hepatitis of unknown etiology in children in the United Kingdom ((1)). Since this information became known, additional cases of acute hepatitis have been reported in multiple countries worldwide. As of July 8, 2022, 35 countries have reported 1,010 probable cases of severe acute hepatitis of unknown etiology in children since October 2021 ((2)). In Canada, acute hepatitis is not a reportable disease therefore, there were no surveillance data available to determine if Canada was experiencing any unusual increases in the number of children presenting with acute hepatitis. Acute hepatitis in children does occur, and frequently the etiology is unknown. It occurs along a spectrum from asymptomatic elevation of liver enzymes to liver failure ((3)). Even without a specific diagnosis, most children recover fully with supportive care. The rarest outcome is fulminant liver failure that necessitates liver transplantation. One approach to determine if there is an increase in acute fulminant hepatitis in children is to collect data on yearly numbers of paediatric liver transplants. This report provides a summary of an investigation of paediatric liver transplantation in Canada.

## Investigation

All paediatric liver transplants in Canada are performed at one of three transplant centres: Stollery Children’s Hospital (SCH, Edmonton, Alberta), The Hospital for Sick Children (HSC, Toronto, Ontario) and “Le Centre hospitalier universitaire Sainte-Justine” (SJ, Montréal, Québec). Data pertaining to the number of liver transplants occurring in children under 16 years of age for paediatric acute liver failure was collected from all three centres. This investigation included cases of acute hepatitis, not attributable to viruses A–E, leading to liver transplant; including those diagnosed with autoimmune hepatitis. Cases with metabolic, genetic, congenital, oncologic, vascular or ischemia conditions or known toxin etiologies that would lead to hepatitis were excluded.

**Figure 1:**
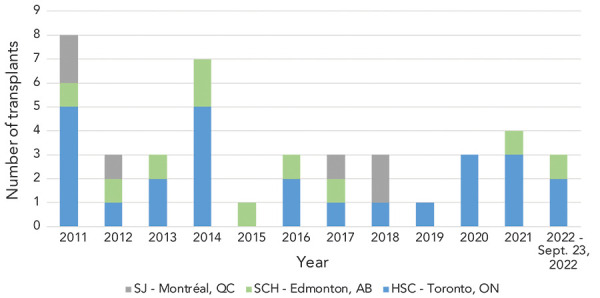
Number of liver transplants in children under 16 years of age due to acute liver failure by transplant centre in Canada, January 2011–September 23, 2022 Abbreviations: AB, Alberta; HSC, Hospital for Sick Children; ON, Ontario; QC, Québec; SCH, Stollery Children’s Hospital; SJ, Le Centre hospitalier universitaire Sainte-Justine

A total of 39 paediatric liver transplants occurred in Canada between 2011 and 2021, with a mean of 3.5 (median: 3; range: 1–8) each year (**Figure 1**). The majority (61.5%) of liver transplants occurred at one institution (HSC). As of August 2022, three liver transplants have been done in 2022.

The national investigation of acute hepatitis of unknown origin focused on cases occurring since October 2021 ((4)). When the investigation was closed on September 23, 2022, a total of four cases of acute hepatitis that resulted in a liver transplant were identified in Canada, one in 2021 and three in 2022.

## Discussion

Based on the available data there was no apparent increase in the number of paediatric liver transplants during the period of interest from October 2021 to September 23, 2022, in Canada. This supported the findings of another Canadian investigation, which did not identify any increase in acute hepatitis of unknown etiology in children ((4)). A federal/provincial/territorial investigation was conducted to actively look for cases of acute hepatitis of unknown origin in children and found no increase in the number of cases of severe acute hepatitis in children.

Since there are only three paediatric transplant centres in Canada, each with specialists who have established working relationships, the Public Health Agency of Canada was able to connect quickly with specialists and determine if they were experiencing an increased need for paediatric liver transplants. This provided a quick mechanism to determine if there were any concerning trends observed in paediatric liver transplants in Canada. Collecting historical baseline data from the transplant centres provided further evidence to support the findings that there had been no increase in paediatric liver transplantation.

There are many causes of paediatric acute liver failure, including infectious agents, metabolic diseases and toxigenic causes; however, in up to 50% of cases, the cause is unknown ((3)). At the preliminary stage of the investigation, it was important to have a broad case definition to ensure that all possible cases were included. Cases with other known hepatotropic viruses (e.g. human herpes virus, Epstein-Barr virus) were also included because of the possibility of co-infection with an unknown or new viral strain. Similarly, cases with autoimmune hepatitis were included because of the possibility that the condition was triggered or exacerbated by a viral infection.

Since initially reporting on an increase in acute hepatitis in children, the investigation in the United Kingdom has developed a hypothesis that cases of acute hepatitis were triggered by co-infection with adenovirus, or human herpesvirus 6B, and adenovirus-associated virus (AAV2) ((5,6)). Investigation in the United States also found cases of co-infection with AAV2 and adenovirus ((7)). Additional research is needed to understand the role of co-infection in the development of acute hepatitis in children.

## Limitations

This study is limited to reporting on trends in the number of paediatric liver transplants in Canada. Detailed medical records of cases were not examined; therefore, the causes of liver transplantation were not analyzed for any trends or commonalities.

## Conclusion

No observable increase in the number of paediatric transplants conducted from October 2021 to September 23, 2022, was seen in the three Canadian transplant centres. Additional research is needed to understand the role of co-infection in the development of paediatric acute hepatitis.

## References

[1] World Health Organization. Disease Outbreak News; Acute hepatitis of unknown aetiology - the United Kingdom of Great Britain and Northern Ireland. Geneva (CH): WHO; April 15, 2022. https://www.who.int/emergencies/disease-outbreak-news/item/acute-hepatitis-of-unknown-aetiology---the-united-kingdom-of-great-britain-and-northern-ireland

[2] World Health Organization. Disease Outbreak News; Severe acute hepatitis of unknown aetiology in children - Multi-country. Geneva (CH): WHO; July 12, 2022. https://www.who.int/emergencies/disease-outbreak-news/item/2022-DON400

[3] Squires RH Jr, Shneider BL, Bucuvalas J, Alonso E, Sokol RJ, Narkewicz MR, Dhawan A, Rosenthal P, Rodriguez-Baez N, Murray KF, Horslen S, Martin MG, Lopez MJ, Soriano H, McGuire BM, Jonas MM, Yazigi N, Shepherd RW, Schwarz K, Lobritto S, Thomas DW, Lavine JE, Karpen S, Ng V, Kelly D, Simonds N, Hynan LS. Acute liver failure in children: the first 348 patients in the pediatric acute liver failure study group. J Pediatr 2006;148(5):652–8. 10.1016/j.jpeds.2005.12.051 16737880 PMC2662127

[4] Macri J, Morton V, Hamel M, Trépanier PL, Salvadori MI. Acute Hepatitis Investigation Team. Acute severe hepatitis of unknown origin in children in Canada. Can Commun Dis Rep 2023;49(6):256–62. 10.14745/ccdr.v49i06a02 38435453 PMC10907059

[5] Ho A, Orton R, Tayler R, Asamaphan P, Tong L, Smollett K, Davis C, Manali M, McDonald SE, Pollack L, Evans C, Menamin J, Roy K, Marsh K, Divala T, Holden M, Lockhart M, Yirrell D, Currie S, Shepherd SJ, Jackson C, Gunson R, MacLean A, McInnes N, Battle R, Hollenback J, Henderson P, Chand M, Hamilton MS. Estrada-Rivadeneyra, Levin M, DIAMONDS consortium, ISARIC4C Investigators, Robertson DL, Filipe A, Willett B, Breuer J, Semple MG, Turner D, Baillie JK, Thomson EC. Adeno-associated virus 2 infection in children with non-AE hepatitis. medRxiv 2022.07.19.22277425. 10.1101/2022.07.19.22277425

[6] Morfopoulou S, Buddle S, Montaguth O, Atkinson L, Guerra-Assunção JA, Storey N, Roy S, Lennon A, Lee JC, Williams R, Williams CA, Tutill H, Bayzid N, Bernal LM, Moore C, Templeton K, Neill C, Holden M, Gunson R, Shepherd SJ, Shah P, Cooray S, Voice M, Steele M, Fink C, Whittaker TE, Santilli G, Gissen P, Brown R, Kaufer BB, Reich J, Andreani J, Simmonds P, Alrabiah DK, Hereza SC; DIAMONDS and PERFORM consortia. Andrade C, Anderson G, Kelgeri C, Waddington SN, Diaz JFA, Hatcher J, De S, Chiozzi RZ, Thalassinos K, Jacques TS, Hoschler K, Talts T, Celma C, Gonzalez S, Gallagher E, Simmons R, Watson C, Mandal S, Zambon M, Chand M, Campos L, Martin J, Thomson E, Ushiro-Lumb I, Levin M, Brown JR, Breuer J. Genomic investigations of acute hepatitis of unknown aetiology in children. medRxiv 2022.07.28.22277963. 10.1101/2022.07.28.22277963

[7] Servellita V, Gonzalez AS, Lamson DM, Foresythe A, Huh HJ, Bazinet AL, Bergman NH, Bull RL, Garcia KY, Goodrich JS, Lovett SP, Parker K, Radune D, Hatada A, Pan CY, Rizzo K, Bertumen JB, Morales C, Oluniyi PE, Nguyen J, Tan J, Stryke D, Jaber R, Leslie MT, Lyons Z, Hedman H, Parashar U, Sullivan M, Wroblewski K, Oberste MS, Tate JE, Baker JM, Sugerman D, Potts C, Lu X, Chhabra P; CDC Pediatric Hepatitis of Unknown Etiology Working Group. Ingram LA, Shiau H, Britt W, Gutierrez Sanchez LH, Ciric C, Rostad CA, Vinge J, Kirking JL, Wadford DA, Raborn RT, St. George K, Chiu CY. Adeno-associated virus type 2 in children from the United States with acute severe hepatitis. medRxiv 2022.09.19.22279829. 10.1101/2022.09.19.22279829

